# How common is ponticulus posticus on lateral cephalograms?

**DOI:** 10.1186/s13104-017-2494-z

**Published:** 2017-04-28

**Authors:** Jamal Giri, Prabhat Ranjan Pokharel, Rajesh Gyawali

**Affiliations:** Department of Orthodontics, College of Dental Surgery, B.P. Koirala Institute of Health Sciences, Dharan, Nepal

**Keywords:** Cervical vertebra, Lateral cephalogram, Ponticulus posticus

## Abstract

**Background:**

Ponticulus posticus is an anomaly of first cervical vertebra visible on lateral cephalogram and has some serious medical and surgical implications. Unfortunately, it is often overlooked or undetected by orthodontists. The general objective of this study is to sensitize orthodontists about this anomaly by depicting its prevalence among a group of Nepalese orthodontic patients.

**Methods:**

Four hundred and fourteen digital lateral cephalograms of orthodontic patients were retrieved from the archives of the department. The lateral cephalograms were carefully assessed for the presence of ponticulus posticus in the posterior spine of atlas vertebra by two investigators independently and the findings were recorded.

**Results:**

Ponticulus posticus was observed in 35.7% of the cases, of which 30.9% had partial ponticulus posticus and 4.8% had complete ponticulus posticus. Even though there was some female predilection, no statistically significant association was found between gender of the patient and presence of ponticulus posticus.

**Conclusion:**

Ponticulus posticus is a fairly common anomaly with more than one-third (35.7%) of a group of Nepalese orthodontic patients affected and is independent of gender. Since, this anomaly is associated with numerous medical conditions and has surgical implications, orthodontists should use lateral cephalogram as screening radiograph for this anomaly.

**Electronic supplementary material:**

The online version of this article (doi:10.1186/s13104-017-2494-z) contains supplementary material, which is available to authorized users.

## Background

The importance of lateral cephalogram in orthodontics cannot be overstated. It has been a lynchpin of orthodontic diagnosis and treatment planning since Down introduced the first cephalometric analysis in the late 1940s. The cervical vertebrae visible in the lateral cephalogram are used for assessing the skeletal maturation of growing patients with skeletal discrepancy for growth modification [1]. Apart from that, the cervical vertebrae are not given due attention during routine orthodontic evaluation of lateral cephalograms. Studies have shown that various cervical vertebral anomalies and pathologies can be detected in lateral cephalograms [2–5]. One of such anomalies that can be discerned by watchful eyes is ponticulus posticus.

The ponticulus posticus is a bony bridge in the first cervical vertebra between the lateral mass and the posterior arch. It results due to ossification of the posterior atlanto-occipital ligament of atlas and encloses the vertebral artery and the first cervical nerve root [6]. It has been reported in literature with different names viz; Kimmerle anomaly [7], Atlas bridging [8], Arcuate foramen [9] etc. The clinical significance of this anomaly is somewhat controversial because studies have found a possible association of this cervical spine anomaly with conditions like Migraine, cervicogenic headache and vertigo but there are patients who are asymptomatic despite having ponticulus posticus [10–12]. However, failure to detect ponticulus posticus can have grave complications during cervical spine surgical intervention, especially those requiring screw placement in lateral mass region of Atlas vertebra [13].

Ponticulus posticus has been investigated for over 100 years now using various methods like evaluation of vertebrae in cadavers, lateral cephalograms and three dimensional (3D) images. But orthodontic literatures about this anomaly are scarce. It is such an irony given that orthodontists are examining lateral cephalograms everyday with the anomaly just around the corner but is often undetected or overlooked. Orthodontists are not directly involved in the management of migraine or neck pain of patients but as health care professionals it is their responsibility to record any anomaly which could be the etiology of such conditions and refer to the concerned physician if needed. Lateral cephalogram can be a screening radiograph for ponticulus posticus only if orthodontists are familiar with this anomaly of the first cervical vertebra as an old adage goes, “the eyes see what the mind knows”.

In the existing literature, a study depicting the prevalence of ponticulus posticus in the Nepalese population is still missing. Hence, the primary objective of the present study is to determine the prevalence of ponticulus posticus among a group of Nepalese orthodontic patients and the secondary objective is to detect any association between the gender of the patient and the presence of this anomaly. This will provide additional population data concerning this anomaly (Additional file 1).

## Methods

Four hundred and fourteen digital lateral cephalograms of the patients presenting for orthodontic treatment were retrieved from the archives of Department of Orthodontics BP Koirala Institute of Health Sciences, Dharan, Nepal. All the radiographs were taken between January 2014 and January 2016. The radiographs of patients with the following characteristics were excluded from the study:

1. Poor display of the posterior arch of the first cervical vertebra due to overlapping of the mastoid process or occiput.
2. Craniofacial syndromes and cleft lip and palate.
3. History of trauma in cervical spine region.

**Fig. 1:**
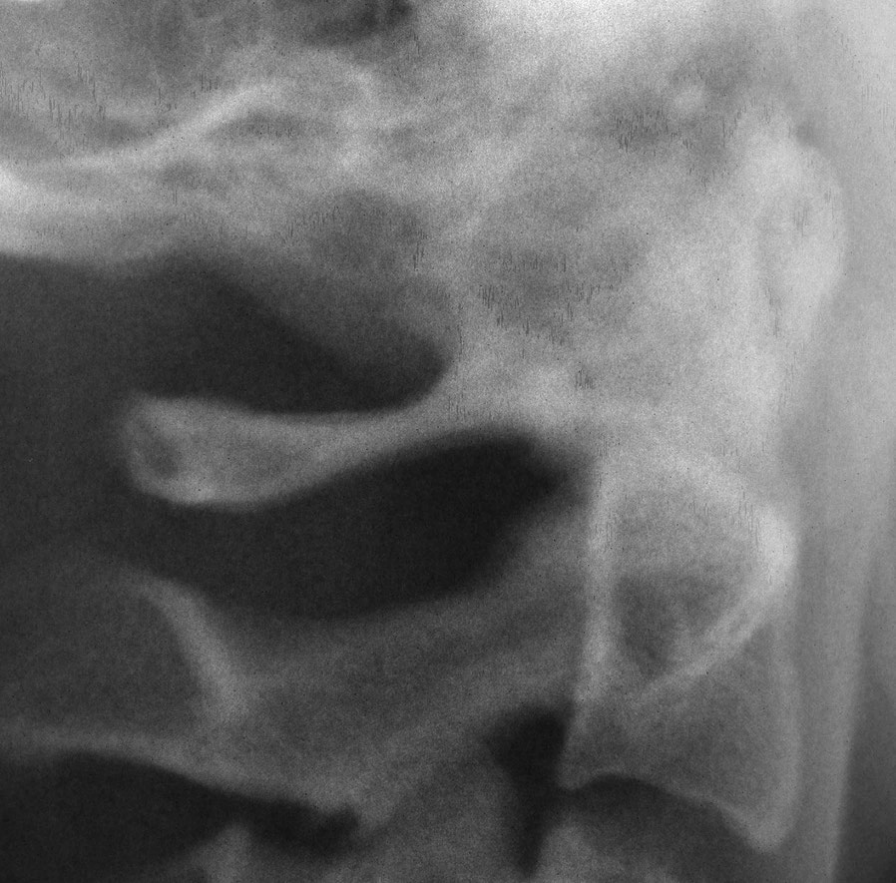
Cropped image of lateral cephalogram with normal cervical vertebra

**Fig. 2:**
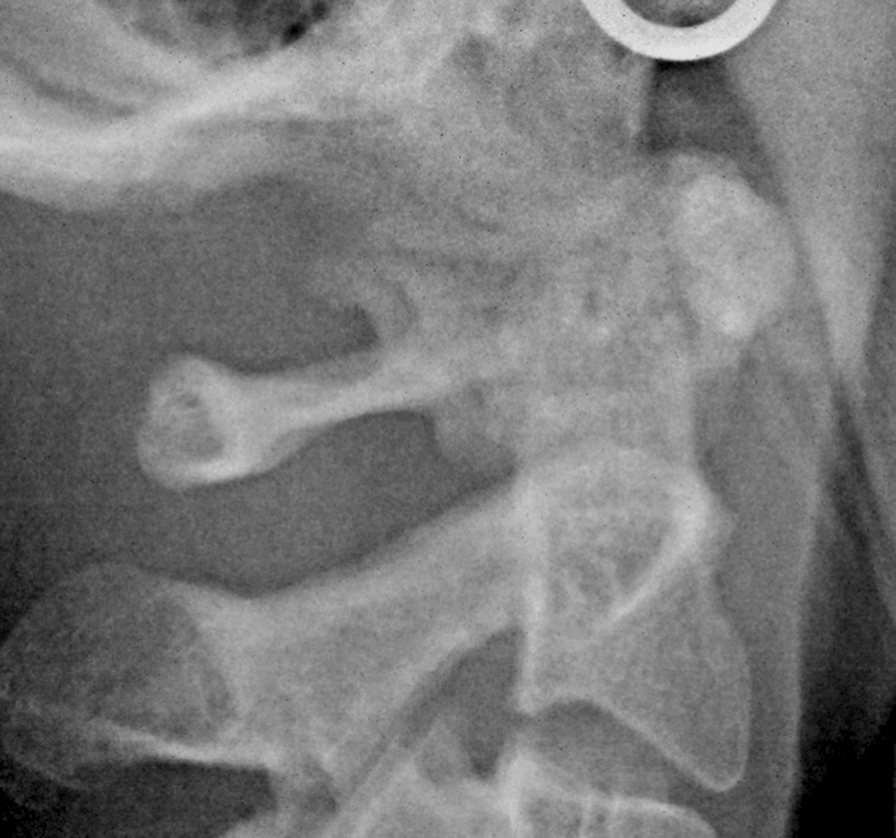
Cropped image of lateral cephalogram with partial ponticulus posticus

**Fig. 3:**
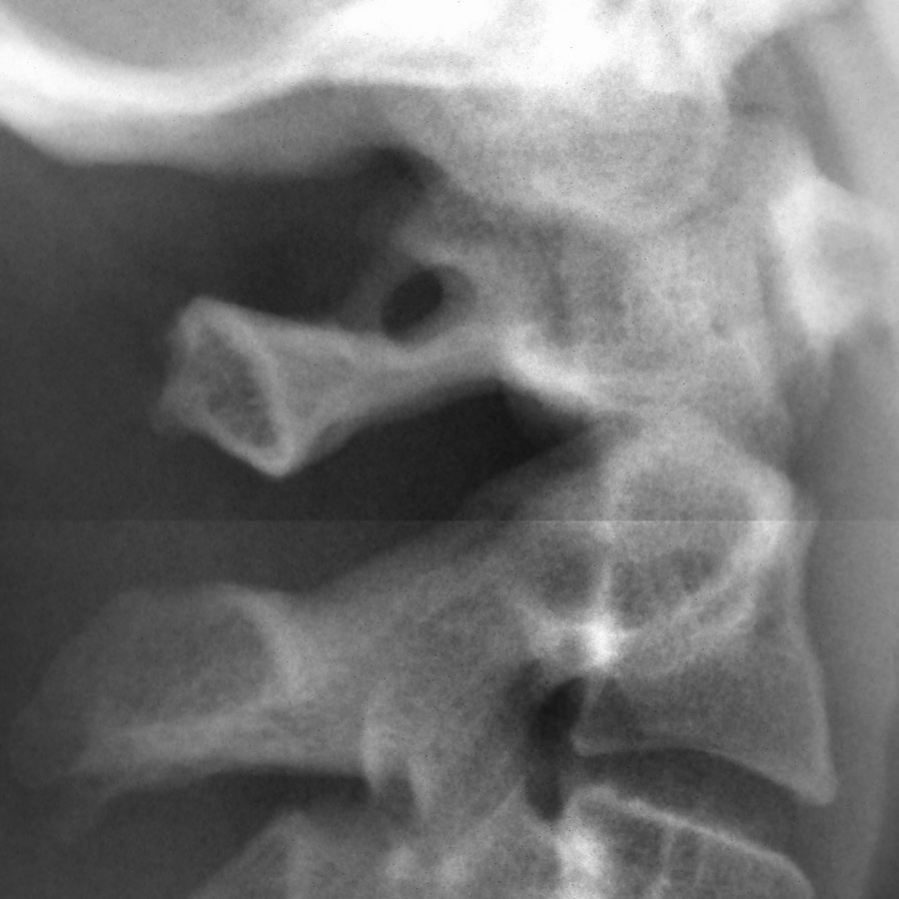
Cropped image of lateral cephalogram with complete ponticulus posticus

The evaluation of digital radiographs was carried out on a computer screen at 1280 × 800 screen resolution. The lateral cephalograms were carefully assessed for the presence of ponticulus posticus in the posterior spine of atlas vertebra by two investigators (JG and RG) independently. Each lateral cephalogram was classified in one of the three ways: absence of ponticulus posticus (Fig. 1), partial ponticulus posticus (Fig. 2) or complete ponticulus posticus (Fig. 3). In case of disagreement between the two investigators a third investigator (PRP) was involved in the decision making process. Final decision was reached on mutual consensus.

SPSS software version 11 was used for data analysis and descriptive statistics were calculated for the samples. Association between the gender of the patient and presence of ponticulus posticus was evaluated using Chi square test with Yates correction.

## Results

The mean age of the samples (246 females and 168 males) was 20.59 ± 4.6 years with a range of 13–41 years. Ponticulus posticus was observed in 35.7% of cases, of which 30.9% had partial ponticulus posticus and 4.8% had complete ponticulus posticus.

The data indicate that this anomaly is higher among females compared to males. 79 females (32.11% of female sample) had partial ponticulus posticus and complete ponticulus posticus was seen in 15 females (6.09%). However, males had partial form of this anomaly in 49 samples (29.16% of male sample) and complete ponticulus posticus in 5 samples (2.9%) (Table 1). But, this difference was not statistically significant (Table 2).

**Table 1:** Distribution of prevalence of ponticulus posticus by gender

Gender	Ponticulus posticus			Total
	Complete	Partial	Absent	
Female	15	79	152	246
Male	5	49	114	168
Total	20	128	266	414

**Table 2:** Chi square test for association of gender and ponticulus posticus

	Value	df	Asymp. Sig. (2-sided)
Pearson Chi square	2.866	2	0.239
Likelihood ratio	2.993	2	0.224
Number of valid cases	414		

## Discussion

In this cross sectional study, lateral cephalograms of a group of Nepalese orthodontic patients were evaluated for the presence of ponticulus posticus. The current study found that the prevalence of ponticulus posticus was 35.7% among a group of Nepalese orthodontic patients with complete ponticulus posticus present in 4.8% of samples. Similar prevalence were reported in studies conducted in England [14] and Northern Greece [15]. However, a number of studies [16–18] have reported a lower prevalence rate of ponticulus posticus than the present study and there are few studies [19–21] reporting higher prevalence as well. These differences can be attributed to the differences in the ethnicity of the samples around the world.

The other reason for this discrepancy in the prevalence rate could be the method by which this anomaly was assessed. Previous studies had utilized lateral cephalograms [17, 20], cadavers [14, 15], computed tomography (CT) images [16, 22] and cone beam computed tomography (CBCT) images [18, 23] to assess the prevalence of this anomaly. Moreover, studies have also shown increased accuracy in diagnosis of ponticulus posticus with three dimensional (3D) imaging [16, 24]. However, lateral cephalogram was used in this study for screening ponticulus posticus because it is a routine radiograph for orthodontic patients.

The result of this study showed that even though there was some female predilection, there was no statistically significant association between gender of the patient and presence of ponticulus posticus. This finding is in agreement with those of previous studies [12, 17, 21, 24, 25]. But, few studies have reported male predilection for this anomaly [6, 22].

There are conflicting reports regarding the association of chronological age of person and presence of ponticulus posticus. Some studies have suggested that this anomaly is a result of senile ossification and has predilection for older age groups [15, 18, 20]. However, other studies did not find a statistically significant association between the chronological age and the presence of ponticulus posticus [12, 23, 26]. This study could not elucidate the relationship between chronological age and this anomaly owing to a narrow range of chronological age of the samples (13–41 years). Further studies with wider range of chronological age of samples and preferably longitudinal studies will help us understand this relationship.

Studies have shown a possible association between this anomaly and conditions like migraine and chronic tension type headache. Some studies have also suggested using ponticulus posticus as one of the major criteria for diagnosis of nevoid basal cell carcinoma syndrome [27, 28]. Furthermore, grave complications await if this anomaly is not identified before lateral mass screw insertion in the first cervical vertebra [13]. Since, ponticulus posticus is fairly common and can be easily detected in lateral cephalograms. Lateral cephalogram should be used as a screening radiograph for this anomaly by orthodontists and if the anomaly is detected it should be documented in patient’s health record for future reference.

## Conclusion

Ponticulus posticus is a fairly common anomaly with more than one-third (35.7%) of a group of Nepalese orthodontic patients affected and is independent of gender. Since, this anomaly is associated with numerous medical conditions and has surgical implications, orthodontists should use lateral cephalogram as a screening radiograph for this anomaly.

## Additional file

**Additional file 1.** Master-table of data.

## Abbreviations

3D: three-dimensional
CT: computed tomography
CBCT: cone beam computed tomography
